# Development of Quaternary InAlGaN Barrier Layer for High Electron Mobility Transistor Structures

**DOI:** 10.3390/ma15031118

**Published:** 2022-01-31

**Authors:** Justinas Jorudas, Paweł Prystawko, Artūr Šimukovič, Ramūnas Aleksiejūnas, Jūras Mickevičius, Marcin Kryśko, Paweł Piotr Michałowski, Irmantas Kašalynas

**Affiliations:** 1 Terahertz Photonics Laboratory, Center for Physical Sciences and Technology (FTMC), Saulėtekio al. 3, LT-10257 Vilnius, Lithuania; arturas.simukovic@ftmc.lt; 2 Institute of High Pressure Physics PAS (UNIPRESS), ul. Sokołowska 29/37, 01-142 Warsaw, Poland; pprysta@unipress.waw.pl (P.P.); krysko@unipress.waw.pl (M.K.); 3 Institute of Photonics and Nanotechnology, Vilnius University, Saulėtekio al. 3, LT-10257 Vilnius, Lithuania; ramunas.aleksiejunas@ff.vu.lt (R.A.); juras.mickevicius@ff.vu.lt (J.M.); 4 Lukasiewicz Research Network-Institute of Microelectronics and Photonics, al. Lotników 32/46, 02-668 Warsaw, Poland; pawel.michalowski@imif.lukasiewicz.gov.pl; 5 Institute of Applied Electrodynamics and Telecommunications, Vilnius University, Saulėtekio al. 3, LT-10257 Vilnius, Lithuania

**Keywords:** InAlGaN, GaN, high electron mobility transistor structures, III-nitride heterostructures, microwave power devices, THz plasmonic devices

## Abstract

A quaternary lattice matched InAlGaN barrier layer with am indium content of 16.5 ± 0.2% and thickness of 9 nm was developed for high electron mobility transistor structures using the metalorganic chemical-vapor deposition method. The structural, morphological, optical and electrical properties of the layer were investigated planning realization of microwave power and terahertz plasmonic devices. The measured X-ray diffraction and modeled band diagram characteristics revealed the structural parameters of the grown In_0.165_Al_0.775_Ga_0.06_N/Al_0.6_Ga_0.4_N/GaN heterostructure, explaining the origin of barrier photoluminescence peak position at 3.98 eV with the linewidth of 0.2 eV and the expected red-shift of 0.4 eV only. The thermally stable density of the two-dimension electron gas at the depth of 10.5 nm was experimentally confirmed to be 1.2 × 10^13^ cm^−2^ (1.6 × 10^13^ cm^−2^ in theory) with the low-field mobility values of 1590 cm^2^/(V·s) and 8830 cm^2^/(V·s) at the temperatures of 300 K and 77 K, respectively.

## 1. Introduction

Currently, III-nitride high electron mobility transistor (HEMT) structures based on the AlGaN/GaN heterostructure are extensively used in various applications such as gas sensors [1,2], THz detectors [3,4,5,6] and emitters [7,8], and high-power microwave devices [9,10,11,12]. A combination of InN and AlN alloys with very different bandgaps, lattice and spontaneous polarization constants can be used to implement a wide range of electric and optic properties in the heterostructure [13,14,15]. In particular, the lattice-matched (LM) InAlN/GaN heterostructures are very attractive for the development of HEMTs as they possess no degradation due to mechanical stress and exhibit high chemical and thermal stability, demonstrated at up to 1000 °C [16,17]. The use of thinner LM-InAlN barrier allows for the achievement of much higher two-dimensional electron gas (2DEG) densities than that in standard AlGaN/GaN HEMT structures with the same barrier thickness [18]. Moreover, barrier-layer scaling in InAlN/GaN HEMT structures down to 3 nm was demonstrated, exhibiting thermal stability (up to 1000 °C) of the barrier interface with a GaN buffer and with surface metals, used for electric contacts [19]. All of the reported features of the LM-InAl(Ga)N/GaN HEMT structures were found to be very attractive for high power GaN-based electronic [11,20,21,22] and plasmonic devices [23,24]. In particular, optimal conditions for the excitation of 2D plasmons in standard AlGaN/GaN HEMT structures up to the room temperature were revealed recently [25]. Among other aspects, a shallow depth and a high density of 2DEG in grating-gated heterostructures allow for efficient plasmonic device operation in the THz regime [26]. In this work, the quaternary LM-InAlGaN barrier layer with indium content of 16.5 ± 0.2% and thickness of only 9 nm was developed for GaN-based heterostructures on sapphire substrate and investigated studying material structural properties, optical and electrical characteristics of the test devices.

## 2. Samples

The heterostructure was developed on 2″ diameter 430 µm thick single-side polished sapphire substrate. The layers with a nominal thickness of 0.5 nm of GaN cap, 8.7 nm of In_x_Al_1–x_N barrier, 1.2 nm of Al_0.66_Ga_0.34_N spacer, 600 nm of undoped GaN channel, and 3.2 µm GaN:C buffer on 25 nm low temperature GaN nucleation layer were grown along the polar *c*-axis using the metalorganic chemical vapor deposition (MOCVD) method in a Close Coupled Showerhead (CCS) Flip Top 3 × 2 inch reactor (Aixtron, Herzogenrath, Germany). A schematic diagram of the heterostructure layers is also discussed in Section 3.3. Of note, if the thickness of the AlN spacer is larger than 1 nm, its role in the formation of 2DEG layer is not as significant due to the polarization discontinuity with the barrier, which was estimated to be of 0.73 eV from the effective conduction band offset between the LM-InAlN and GaN [18]. Moreover, the target of a ~9 nm thickness of the barrier was chosen as optimum for high values of 2DEG density and mobility [19], with an expectation for higher transconductance values due to the short distance between the transistor gate and a 2DEG channel.

**Figure 1:**
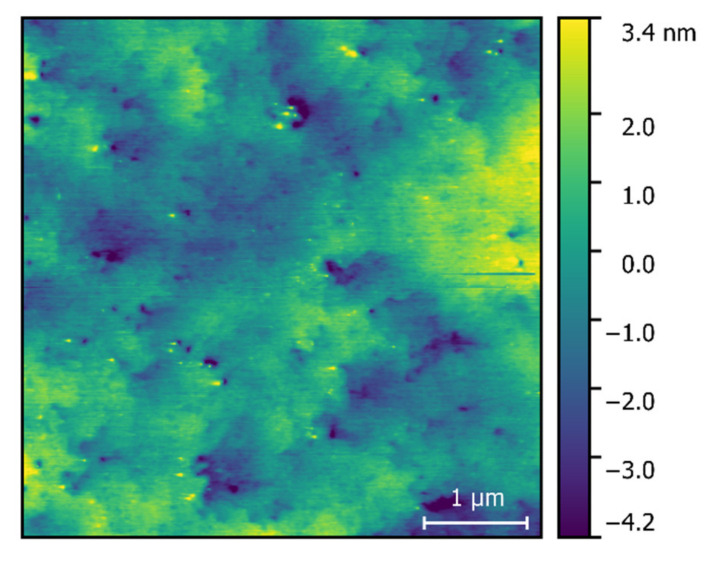
AFM image of the as-grown heterostructure.

The AFM image of an as-grown heterostructure was measured with Dimension 3100 AFM instrument (Veeco, TX, USA). The results are shown in Figure 1. The RMS roughness over the whole image area (4 × 4 µm^2^) was found to be 1.2 nm. The threading dislocation density (TDD) was estimated over the area of 2 × 2 µm^2^, counting the pits, which are seen as the black color points in Figure 1. The value of TDD was around 2 × 10^9^ cm^−2^.

The sheet resistance was measured by the microwave single-post dielectric resonator QWED SiPDR (QWED, Warsaw, Poland). At room temperature, the as-grown heterostructure demonstrated sheet resistance values of 350 ± 1 Ω/sq.

Mesas for test devices were fabricated using inductively coupled plasma reactive ion etching (ICP-RIE) (Oxford Instruments, Bristol, UK) via Cl plasma. For ohmic contacts, Ti/Al/Ni/Au metal stack of thicknesses 30/90/20/150 nm were deposited and annealed in nitrogen ambient for 30 s at 850 °C. Schottky contacts were formed of 25/150 nm Ni/Au stack. The set of the test devices consisted of Van der Pauw (VdP) structures, transmission line method (TLM) resistor array, HEMTs and Schottky barrier diodes (SBDs). Furthermore, test devices were left with an unpassivated nitride surface. More details about the device processing have been reported elsewhere [12,27].

Test TLM structures were used to estimate the resistance of ohmic contacts and the sheet resistance of the 2DEG layer. Their values were found to be of 1 Ω mm and 385 Ω/sq., respectively. The ideality factor and Schottky barrier height were determined from forward biased current-voltage characteristics of the SBDs applying the thermionic emission model [28], demonstrating the values of about 2.3 and 0.7 eV, respectively.

## 3. Results

### 3.1. Secondary-Ion Mass Spectrometry (SIMS) of the Heterostructure

A SIMS analysis of as-grown heterostructure was performed in order to determine impurity density and distribution of atomic fractions in the layers. In this work SIMS depth profiles were performed employing the SC Ultra instrument (CAMECA, Gennevilliers, France) under ultra-high vacuum. The amount of residual oxygen, carbon, and silicon impurities in the GaN channel layer were found from the calibrated SIMS data to be of (3.7 ± 1.3) × 10^16^ cm^−3^, (5.1 ± 0.3) × 10^16^ cm^−3^, and (1.0 ± 0.6) × 10^16^ cm^−3^, respectively. The amount of these impurities was close to that obtained by the low-frequency noise and THz electroluminescence spectroscopy methods in the AlGaN/GaN HEMT on sapphire [27,29].

**Figure 2:**
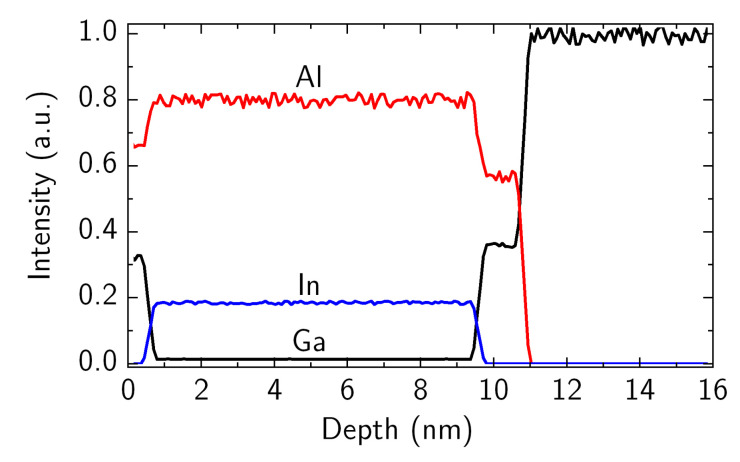
SIMS profile of Ga, In, and Al in the heterostructure under study.

Figure 2 demonstrates the very uniform distribution of each atomic fraction along the growth direction of the heterostructure layers. A small fraction of Ga was found in the barrier, which occasionally occurs during the growth of the InAlN barrier in MOCVD reactor [30,31,32]. However, the CCS tools are known to accumulate the gallium-containing material on the inlet showerhead flange upstream the substrates. This deposit can be the unintentional source of the gallium incorporated into the main barrier layer grown and enhanced by TMIn and hydrogen carrier gas delivery [30].

### 3.2. X-ray Diffraction (XRD) Analysis of the Heterostructure

**Figure 3:**
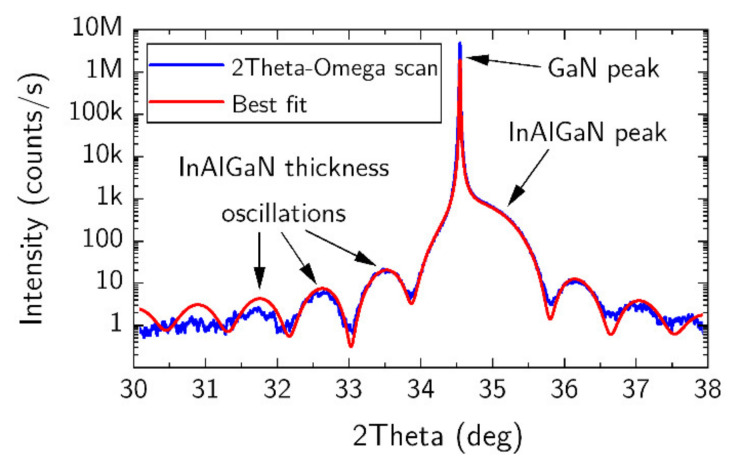
Experimental HR-XRD curve (blue line) and the best fit (red line) of the heterostructure with quaternary barrier composition of 16.5 ± 0.2%, 77.5 ± 0.2%, and 6% for In, Al, and Ga, respectively, and thickness of 8.6 nm.

The crystal quality, layer thicknesses and composition of as-grown heterostructure were investigated by HR-XRD analysis (Malvern Panalitycal X’pert MRD, Almelo, Netherlands). The XRD results of as-grown heterostructure are shown in Figure 3. With the assumption that the Ga concentration was 6%, other concentrations were found to be of 16.5 ± 0.2% In (error coming from fitting of the simulation) and 77.5 ± 0.2% Al. To calculate the fitted curve the dynamical diffraction theory was used. Errors coming from uncertainty of elastic constants were negligible as the layer was not strained. Additionally, lattice constant, *a*, of the quaternary barrier was calculated with Nextnano++ software (Nextnano GmbH, München, Germany). The lattice matching to GaN with deviation of $a_{\Delta }=((a_{Barier}-a_{GaN})/a_{GaN})\times 100\%$ was found to be as small as −0.03%. Moreover, a single layer of 30 nm thick InAl(Ga)N was grown on the GaN template using the same growth conditions as for the investigated heterostructure. The XRD experiment confirmed that the barrier layer is lattice matched to GaN.

It is worth discussing the algorithm used in our work for the development of the LM barrier layers. Our method to assess the InAlN (In = 18%, AlN = 82%) barrier composition the path was as follows:

(i). we made expected calculations of the gas-phase with surplus indium and aluminum precursors only, without flowing the gallium,
(ii). we grew 30–50 nm thick calibration samples on GaN/Sapphire increasing the growth temperature and checking for lattice-matching to GaN in-plane lattice compatibility by HR-XRD,
(iii). we applied the preferred high growth temperature and the growth rate to the HEMT final structure, growing a 9 nm thick LM-InAlGaN barrier.

We assumed that some gallium was incorporated into the barrier as expected from similar studies in literature while using the CCS reactors [30]. Therefore, the density of 2DEG and the band gap of barrier layer were experimentally investigated and simulated using Nextnano++ software (see details in next sections). Based on the Nextnano++ results, much higher 2DEG density for nominal ternary composition was expected, therefore, in simulation of HR-XRD data, we iteratively added Ga to the barrier, removing some In and Al but keeping the original Al/In ratio. In this way, the polarization influence on actual 2DEG concentration was decreased until a reasonable agreement between simulation and measurement values were obtained. 

### 3.3. Band Diagram of the Heterostructure

**Figure 4:**
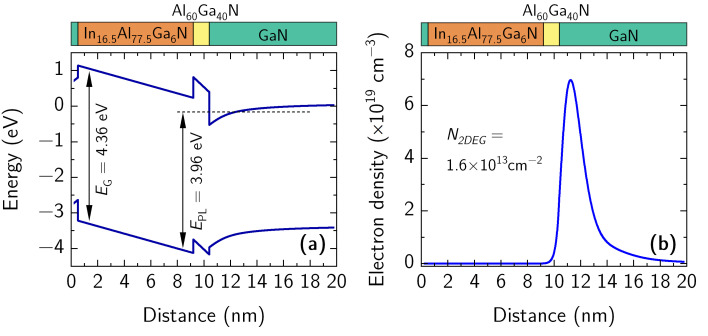
Band diagram (**a**) and 2DEG distribution (**b**) in the heterostructure calculated using the barrier composition determined from XRD measurements. A schematic representation of the heterostructure layout is provided above the figures.

Nextnano++ software was used to calculate the bandgap, lattice constant, 2DEG density in the heterostructure using the layers thicknesses from the growth protocol and quaternary barrier composition from the XRD analysis. The results of modeling are shown in Figure 4. It has been shown that for the best lattice-matching the Al/In ratio should be 4.63 [33,34]. The composition of the modeled quaternary heterostructure demonstrated a very similar Al/In ratio of 4.70. Furthermore, its bandgap of 4.36 eV (Figure 4a) was found to be very close to that of ternary In_x_Al_1–x_N barrier with an In fraction of 18% with *E*_G_ = 4.39 eV (not shown). Finally, the calculated 2DEG density in the quaternary heterostructure was found to be of about 1.6 × 10^13^ cm^−2^.

### 3.4. Two-Dimensional Electron Gas (2DEG)

The carrier density, mobility, and sheet resistance of the 2DEG were determined in Hall experiment using Van der Pauw structures at two temperatures, 77 K and 300 K. The sheet carrier density at both temperatures was 1.2 × 10^13^ cm^−2^, with an uncertainty of 3%. Meanwhile, the mobility increased with the decrease in temperature from 1590 cm^2^ V^−1^ s^−1^ at 300 K to 8830 cm^2^ V^−1^ s^−1^ at 77 K, resulting in the change in sheet resistance from 320 Ω/sq. to 60 Ω/sq., respectively. A small difference in the 2DEG density between calculated 1.6 × 10^13^ cm^−2^ and measured 1.2 × 10^13^ cm^−2^ values can be explained by the heterostructure material sensitivity to the device fabrication steps such as chemical treatment, ion plasma etching, metal electrode deposition, and non-controlled surface unintentional passivation [12,27].

### 3.5. Schottky Contact to 2DEG 

**Figure 5:**
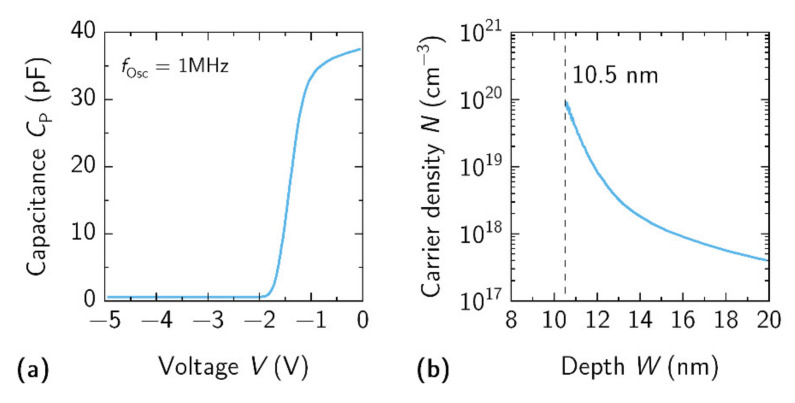
(**a**) Capacitance-voltage (at 1 MHz) measurements for circular SBD with Ø80 µm inner Schottky contact pad which is separated from outer ohmic contact by 5 µm. (**b**) Measured distribution of carrier density in the heterostructure. The position of 2DEG was found at 10.5 nm as marked by dashed vertical line.

The capacitance-voltage (C-V) characteristics of the fabricated Schottky contacts to the 2DEG layer were measured on the EPS150 probe station (Cascade Microtech, Beaverton, OR, USA) using precision impedance analyzer Agilent 4294A (Agilent, Santa Clara, CA, USA). Typical C-V characteristic of the circular SBD with 5 µm separation between ohmic-Schottky contacts and 80 µm diameter of inner Schottky pad are shown in Figure 5a. Data linear extrapolation to abscissa allowed us to determine the pinch-off voltage, *V*_p–o_, which was found at −1.74 V. The C-V results allowed us to estimate the dependence of carrier density, *N*, on the distance from the surface, W, employing the method described elsewhere [35]. The position of the 2DEG under the Schottky contact was found to be 10.5 nm from surface, which is very close to the nominal value of 10.4 nm defined from the growth protocol. It is worth noting that the high 2DEG density was achieved with a barrier thickness of only 9 nm. This is less than a half of the AlGaN barrier layer thickness, which usually varies from 20 to 25 nm in standard AlGaN/GaN HEMT structures [12,27].

### 3.6. Photoluminescence of the Heterostructure

**Figure 6:**
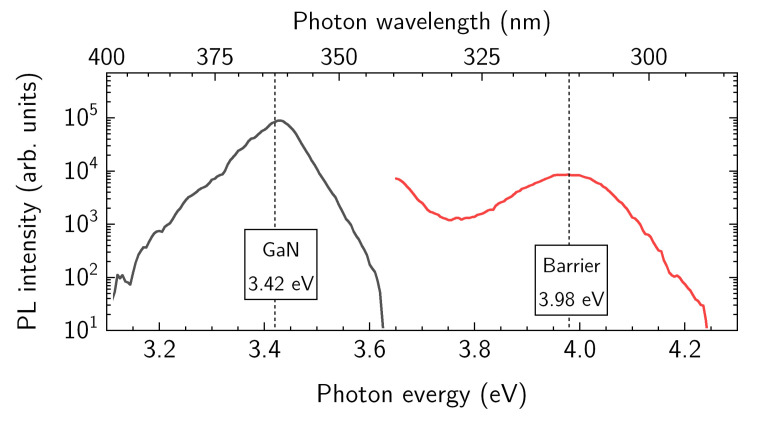
PL spectra of the heterostructure measured using different excitation wavelength and peak power density of 300 nm and 200 kW/cm^2^ (black line) and 250 nm and 3 MW/cm^2^ (red line), respectively.

The photoluminescence (PL) spectra of as-grown heterostructure were measured using an optical parametric oscillator (OPO, Ekspla NT342B, Vilnius, Lithuania) for the excitation, generating four ns-pulses with peak power density of about 200 kW/cm^2^ and 3 MW/cm^2^ at the selected wavelength of 300 nm and 250 nm, respectively. Measured PL spectra are shown in Figure 6. At the excitation wavelength of 300 nm (black line in Figure 6), electron recombination in GaN barrier was probed only, demonstrating a Lorentz-shape band at the position of 3.42 eV with full width at half maximum (FWHM) of 90 meV. At the excitation wavelength of 250 nm, the broad band centered at around 3.98 eV with FWHM = 200 meV was measured in the PL spectrum (red line in Figure 6), revealing the contribution of InAlGaN barrier [36,37].

The results of the PL measurements (see Figure 6) demonstrate a 0.40 eV red-shift from the expected bandgap energy of the quaternary barrier (see Figure 4a). Such a red-shift of the PL peak was sufficiently small in comparison to the Stokes shifts reported for similar heterostructures with InAlN barrier exhibiting values in the range of 0.4–1 eV [36,37,38,39,40]. Various explanations of the Stokes shift were proposed such as the introduction of sub-band edge states near the band edge minima which induce strain and related alloy composition fluctuations [41], local variations of the number of Al and In atoms surrounding nitrogen atom [42] or radiative recombination between the electrons in the triangular quantum well (2DEG) and photoexcited holes efficiently transferred from InAlN to GaN via sub-band-gap states [37].

Taking into account the energy levels of the barrier conduction band in the vicinity of Al_0.66_Ga_0.34_N spacer (237 meV) and of 2DEG (−162 meV) with respect to the barrier bandgap (4.36 eV), the modified bandgap was estimated to be of about *E*_PL_ = 3.96 eV, which is close to the measured PL peak position at 3.98 eV. Additionally, the evaluation of the transition energies in *c*-plane grown heterostructure is somehow affected by carrier confinement and the built-in electric field. Therefore, the developed quaternary heterostructure requires a more detailed investigation of photo-carrier transport and a direct comparison of PL spectra with the results of other methods able to determine the bandgap of barrier layer such as a photoluminescence excitation (PLE), cathode photoluminescence (CL), and reflection modulated spectroscopy.

### 3.7. Application of the Heterostructure for Electronic Devices 

**Figure 7:**
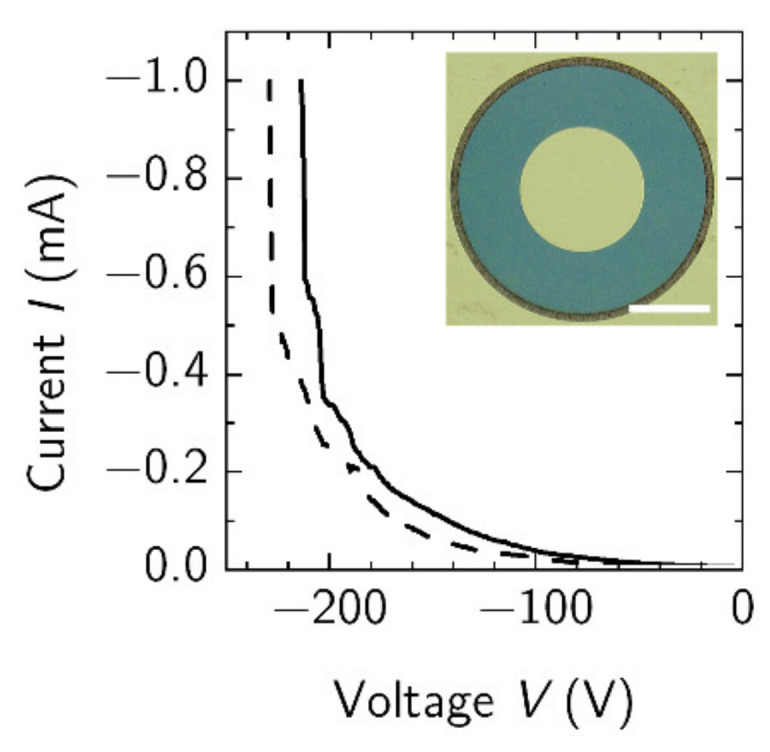
Breakdown of two SBDs with contact spacings of 5 µm (solid line) and 40 µm (dashed line). Current was limited to 1 mA level. Inset–microscope image of the SBD with diameter of central Schottky contact and distance to ohmic contact being of 80 µm and 40 µm, respectively.

The SBDs were processed without surface passivation and investigated in the reverse bias regime in order to find the breakdown fields. The results are shown in Figure 7. For the 5 µm contact separation, SBD breakdown occurred at the electric field of 0.43 MV/cm (Figure 7 solid line). For the largest contact separation of 40 µm, the breakdown field value decreased to 0.05 MV/cm (Figure 7 dashed line). The breakdown of the SBD was found to occur via the surface defects and threading dislocations, both of which create shunting paths for the leakage current, the value of which before the breakdown (at (−200)–(−230) V) was up to 2.14 mA/mm. Furthemore, the SBDs with larger contact separation had a larger active area, which includes more defect and threading dislocations [43,44,45,46]. We would think that large leakage currents could cause a thermal breakdown of the device.

The HEMTs were also processed and studied on the wafer in DC and RF regimes using the Süss Microtech probe station PM8 (FormFactor Inc., Livermore Inc., Livermore, CA, USA). For the RF measurements the G-S-G (ground–signal–ground) high frequency probes, an E8364B PNA Network Analyzer (Keysight Technologies, Santa Rosa, CA, USA), and E5270B Precision IV Analyzer with IC-CAP software were used (Keysight Technologies, Santa Rosa, CA, USA). The two-step open-short de-embedding method was implemented, and small signal S-parameters were obtained. The unity current gain–cut-off frequency (*f*_T_) and the unity maximum unilateral power gain frequency (*f*_max_) were found from de-embedded S-parameter frequency characteristics.

**Figure 8:**
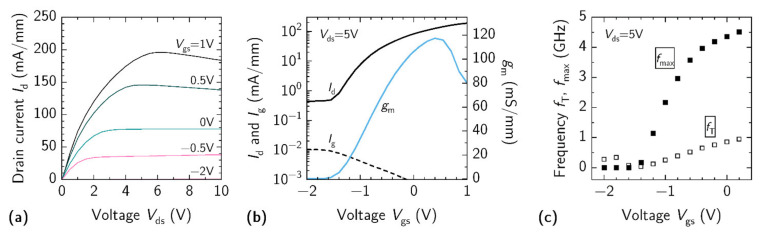
(**a**) Output characteristics, (**b**) transfer characteristics and (**c**) *f*_T_ and *f*_max_ frequencies measured for the same HEMT with *L*_g_ = 5 µm and *L*_ds_ = 12 µm.

Each HEMT was developed in G-S-G device configuration with *W*_Ch_ = 2 µm × 200 µm, *L*_ds_ = 12 µm and *L*_g_ = 5 µm. The results for HEMTs characterization in DC and RF regimes are shown in Figure 8. Output characteristics demonstrate (see Figure 8a) the effect of very low pinch-off voltage, where the channel is fully closed at *V*_gs_ = −2 V. The highest output drain current was recorded at *V*_ds_ = 6 V and *V*_gs_ = 1 V, reaching up to 200 mA/mm, which deteriorate with further increase in drain voltage due to excess heat accumulation in the conductive channel. The transfer curve for the same device at *V*_ds_ = 5 V is shown in Figure 8b. Large leakage currents were observed both in the drain and gate contacts under negative gate bias. The *I*_on_/*I*_off_ ratio for the HEMT was limited to the value of 26 dB. The small-signal transconductance values for this device were measured to be up to 117 mS/mm at *V*_gs_ = +0.6 V. The results of RF characterization are shown in Figure 8c. The highest *f*_T_ and *f*_max_ values at *V*_ds_ = 5 V and *V*_gs_ = 0.2 V were estimated to be up to 0.95 GHz and 4.5 GHz, respectively. The figure of merit value *f*_T_ × *L*_g_ for the investigated HEMT was of 4.3 GHz × µm, which is about two times lower than 10 GHz × µm, reported for the scaled HEMTs made of similar InAl(Ga)N/GaN heterostructures [10,47]. The difference can be explained by a simplified design of our transistors due to the usage of standard UV photolithography instead of e-beam lithography.

## 4. Conclusions

A quaternary LM InAlGaN barrier layer with an indium content of 16.5 ± 0.2% and thickness of only 9 nm has been developed for high electron mobility transistor structures by metalorganic chemical vapor deposition method. A detailed analysis of the structural, morphological, optical and electrical properties revealed the composition of the barrier layer to be 16.5 ± 0.2% of In, 77.5 ± 0.2% of Al, and 6% of Ga, and allowed us to explain the origin of barrier photoluminescence peak position observed at 3.98 eV with the linewidth of 0.2 eV and the expected red-shift of 0.4 eV only. The thermally stable density of the two-dimension electron gas at the depth of 10.5 nm was experimentally confirmed to be of 1.2 × 10^13^ cm^−2^ (1.6 × 10^13^ cm^−2^ in theory) with low-field mobility values of 1590 cm^2^/(V·s) and 8830 cm^2^/(V·s) at the temperatures of 300 K and 77 K, respectively. The fabricated Schottky barrier diodes allowed us to determine the pinch-off voltage to be −1.74 V and breakdown field to be 0.4 MV/cm for this heterostructure. The HEMTs fabricated of this heterostructure were tested both in dc and rf regimes and show similar performance to the standard AlGaN/GaN HEMTs, considering that a substantially thinner barrier layer was used. Overall, the results presented in our work indicate that the developed heterostructure with quaternary LM-InAlGaN barrier is a promising candidate for microwave power electronic and plasmonic devices operating in the THz range.

## Data Availability

The data presented in this study are available on request from the corresponding author. The data are not publicly available due to privacy.
